# Tumor Expression Profile Analysis Developed and Validated a Prognostic Model Based on Immune-Related Genes in Bladder Cancer

**DOI:** 10.3389/fgene.2021.696912

**Published:** 2021-08-27

**Authors:** Bingqi Dong, Jiaming Liang, Ding Li, Wenping Song, Shiming Zhao, Yongkang Ma, Jinbo Song, Mingkai Zhu, Tiejun Yang

**Affiliations:** ^1^Department of Urology, Affiliated Cancer Hospital of Zhengzhou University, Henan Cancer Hospital, Zhengzhou, China; ^2^The Second Affiliated Hospital of Guangzhou Medical University, Guangzhou, China; ^3^State Key Laboratory of Respiratory Disease, The First Affiliated Hospital of Guangzhou Medical University, National Clinical Research Center for Respiratory Disease, Guangzhou, China; ^4^Department of Pharmacy, Affiliated Cancer Hospital of Zhengzhou University, Henan Cancer Hospital, Zhengzhou, China

**Keywords:** bladder cancer, immune-related signature, The Cancer Genome Atlas, Gene Expression Omnibus, immunotherapy

## Abstract

**Background**: Bladder cancer (BLCA) ranks 10th in incidence among malignant tumors and 6th in incidence among malignant tumors in males. With the application of immune therapy, the overall survival (OS) rate of BLCA patients has greatly improved, but the 5-year survival rate of BLCA patients is still low. Furthermore, not every BLCA patient benefits from immunotherapy, and there are a limited number of biomarkers for predicting the immunotherapy response. Therefore, novel biomarkers for predicting the immunotherapy response and prognosis of BLCA are urgently needed.

**Methods**: The RNA sequencing (RNA-seq) data, clinical data and gene annotation files for The Cancer Genome Atlas (TCGA) BLCA cohort were extracted from the University of California, Santa Cruz (UCSC) Xena Browser. The BLCA datasets GSE31684 and GSE32894 from the Gene Expression Omnibus (GEO) database were extracted for external validation. Immune-related genes were extracted from InnateDB. Significant differentially expressed genes (DEGs) were identified using the R package “limma,” and Gene Ontology (GO) analysis and Kyoto Encyclopedia of Genes and Genomes (KEGG) enrichment analysis for the DEGs were performed using R package “clusterProfiler.” Least absolute shrinkage and selection operator (LASSO) regression analysis were used to construct the signature model. The infiltration level of each immune cell type was estimated using the single-sample gene set enrichment analysis (ssGSEA) algorithm. The performance of the model was evaluated with receiver operating characteristic (ROC) curves and calibration curves.

**Results**: In total, 1,040 immune-related DEGs were identified, and eight signature genes were selected to construct a model using LASSO regression analysis. The risk score of BLCA patients based on the signature model was negatively correlated with OS and the immunotherapy response. The ROC curve for OS revealed that the model had good accuracy. The calibration curve showed good agreement between the predictions and actual observations.

**Conclusions**: Herein, we constructed an immune-related eight-gene signature that could be a potential biomarker to predict the immunotherapy response and prognosis of BLCA patients.

## Introduction

Bladder cancer (BLCA) ranks 10th in incidence among malignant tumors and 6th in incidence among malignant tumors in males (Bray et al., 2018). The disease may present as muscularly invasive bladder cancer (MIBC), non-muscularly invasive bladder cancer (NMIBC) or as a metastatic form of other diseases (Tran et al., 2021). With the development of technologies such as electrosurgery, chemotherapy, and radical surgery, the overall survival (OS) rate of BLCA patients has greatly improved. However, primary BLCA are prone to recurrence after systematic treatment, the prognosis is not satisfactory (Rouprêt et al., 2021), and there are no clinically meaningful diagnostic markers. Based on the fact that the incidence of BLCA is gradually increasing, valuable biomarkers are particularly urgently needed.

Current studies have revealed that the tumor microenvironment is closely correlated with tumorigenesis, progression and prognosis, and immune cells of the tumor microenvironment exhibit complex interactions with tumor cells (Hanahan and Weinberg, 2011). As the landmark developments of immune checkpoint inhibitors (represented by anti-PD-1/PD-L1 antibodies), Immunotherapy is playing an increasingly important role in the treatment of BLCA (Powles et al., 2014; Pettenati and Ingersoll, 2018; Rouanne et al., 2018). Thus, immune molecules associated with the tumor microenvironment have a tremendous role in serving as prognostic markers for BLCA. Previous studies have proposed immune-related biomarkers in thyroid and ovarian cancers for risk stratification and clinical outcome prediction (Kim et al., 2018; Shen et al., 2019). A few studies have been conducted to assess the potential of immune-related genes to predict clinical outcomes and the immunotherapy response in BLCA (Qiu et al., 2020; Zhu et al., 2020; Lv et al., 2021), but the depth and results of these studies are not satisfactory.

Herein, the purpose of this study is to find suitable biomarkers of BLCA with high sensitivity and strong specificity and molecular targets that affect the clinicopathological process of BLCA, then provide an important reference for the diagnosis of BLCA. We identified immune-related DEGs for BLCA, and constructed an immune-related eight-gene signature model. The signature model showed good prognostic value for predicting OS and could be used to predict the immunotherapy response in BLCA patients.

## Materials and Methods

### Data Collection and Preprocessing

The RNA sequencing (RNA-seq) data, probe annotation files and clinical data of the BLCA patients were extracted from The Cancer Genome Atlas (TCGA) and used to acquire the expression profiles of the BLCA patients. After screening, samples with no clinical data were excluded. A total of 406 tumor samples and 18 normal samples were included in the analysis. BLCA cohorts GSE31684 and GSE32894 were obtained from the Gene Expression Omnibus (GEO) database using the R package “GEOquery” (Davis and Meltzer, 2007). Immune-related genes were obtained from InnateDB.^1^

### Identification of Differentially Expressed Genes and Functional Annotation

Significant DEGs between normal and BLCA samples were identified with screening criteria of adjusted value of *p*<0.05 and |log fold change (FC)|>1 by the R package “limma” (Ritchie et al., 2015). Gene Ontology (GO) analysis and Kyoto Encyclopedia of Genes and Genomes (KEGG) enrichment analysis for the DEGs were performed using R package “clusterProfiler” (Yu et al., 2012).

### Estimation of the Infiltration Degree of Each Immune Cell in BLCA

A group of immune cell gene markers, consisting of 782 genes, which represent 28 immune cell types related to innate and adaptive immunity, were obtained from previous studies to estimate the infiltration level of different immune cell types in the tumor microenvironment (Charoentong et al., 2017). The assessed immune cell types included, B cells, natural killer (NK) cells, dendritic cells, myeloid-derived suppressor cells (MDSCs), neutrophils, and T cells. Subsequently, the expression profiles of each sample were used to estimate the infiltration level of each immune cell type in BLCA using the single-sample gene set enrichment analysis (ssGSEA) algorithm with the R package “GSVA” (Hänzelmann et al., 2013).

### Survival Analysis

Univariate Cox proportional hazards regression analysis was carried out to evaluate the association between the expression level of the immune-related DEGs and the OS of BLCA patients. Immune-related DEGs with a value of *p*<0.001 based on the log-rank test were selected as candidate genes for construction of the prognostic model. The risk score of each patient was calculated based on the signature model and was used to evaluate the association between the gene signature model and the prognosis of BLCA patients. The samples were assigned to the high-risk or low-risk group based on the median risk score. Kaplan–Meier curves and log-rank tests were performed to compare the differences in OS and progression-free survival between the high-risk and low-risk groups. A statistically significant difference was defined as *p*<0.05. Survival analysis and log-rank tests were performed using the R package “survival,” and the R package “survminer” was used to plot the Kaplan–Meier curve.

### Establishment and Evaluation of the Immune-Related Signature Model

The TCGA BLCA cohort was randomly divided into a training set (*n*=285) and a testing set (*n*=121) at a ratio of 7:3. The R package “glmnet” was used to perform LASSO regularization to reduce the coefficients from the training set. An immune related eight-gene model was constructed. The following formula was used:

$$\begin{array}{c} Riskscore=-0.15744\times DCHS1+0.05310\\ \;\times PTGIS-0.32469\times PTPN6\\ \;-0.07170\times AIFM3+0.11454\\ \;\times FLRT2+0.04565\times PCSK5\\ +0.15854\times CLSTN2-0.11663\times HSH2D \end{array}$$

The risk score was calculated for each sample using the signature model. The BLCA cohort was assigned to high- and low-risk groups based on the risk score. Receiver operating characteristic (ROC) curves for 1-, 3-, and 5-year OS were generated for the two groups using the R package “survivalROC.” Calibration curves were derived from the R package “rms” to evaluate the precision of the 1-, 3-, and 5-year OS prediction. Decision curve analysis was performed using the R package “ggDCA” to quantify the net benefits at different threshold probabilities and evaluate the clinical usefulness of the immune-related signature model.

### Prediction of the Immunotherapy Response

The Tumor Immune Dysfunction and Exclusion (TIDE) algorithm^2^ was used to estimate the response of each sample to anti-PD-1/PD-L1 and anti-CTLA4 immunotherapy based on the gene expression profiles of the BLCA cohort (Jiang et al., 2018).

### Statistical Analysis

Univariate survival analysis was performed using the log-rank test. The Pearson correlation formula was used to calculate the correlations between the risk score and immune markers, the risk score and characteristic gene expression, characteristic gene expression and the immune cell infiltration score, and the risk score and immune cell infiltration score. Two-tailed Student’s *t* tests were used for two-group comparisons. A statistically significant difference was defined as *p*<0.05. All statistical analyses were performed in R version 4.0.2.

## Results

### Identification of Immune-Related DEGs in BLCA

**Figure 1:**
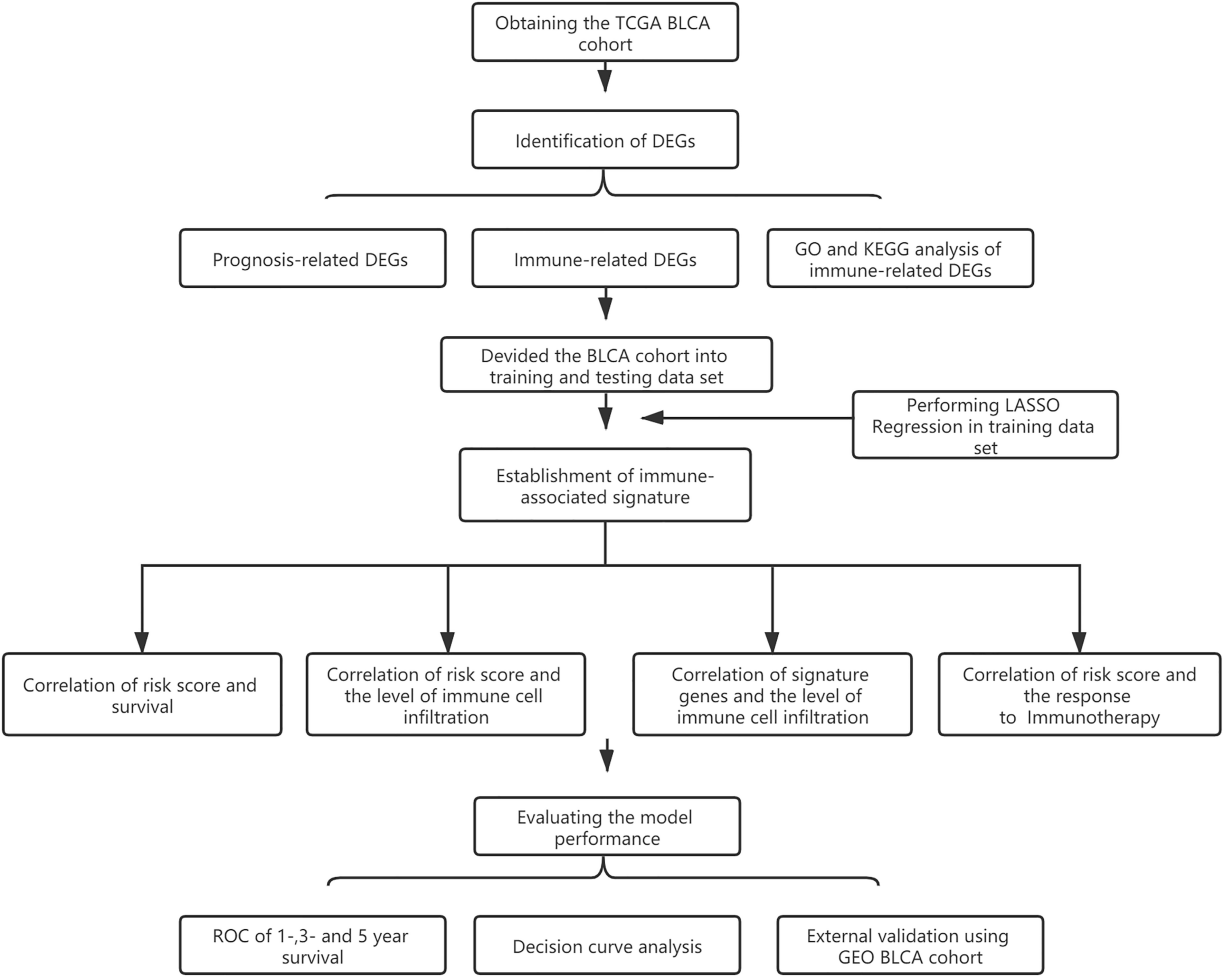
Flow diagram showing the design of the study.

**Figure 2:**
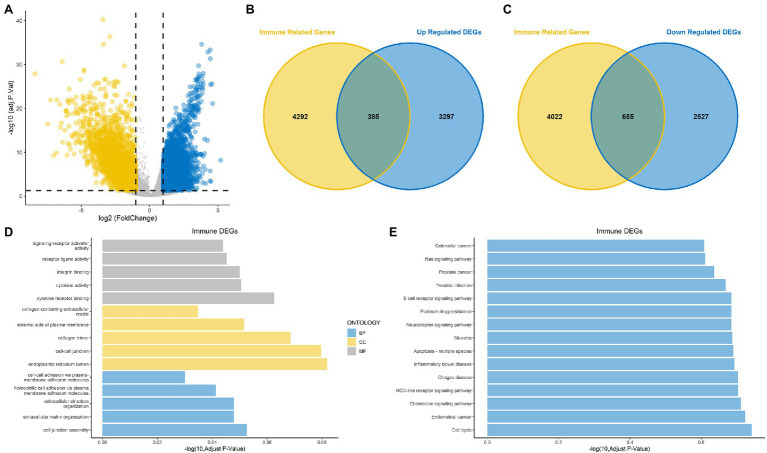
Identification of differentially expressed genes (DEGs) and immune-related DEGs for bladder cancer (BLCA). **(A)** Volcano plot for DEGs for BLCA. **(B,C)** Venn diagram showing up-regulated and down-regulated immune-related DEGs. **(D)** Gene Ontology (GO) functional annotation for immune-related DEGs. **(E)** Kyoto Encyclopedia of Genes and Genomes (KEGG) enrichment analysis for immune-related DEGs.

The overall design is shown in Figure 1. First, to identify DEGs in BLCA, a total of 406 BLCA samples and 18 normal samples were downloaded from the University of California, Santa Cruz (UCSC). A total of 3,677 up-regulated and 3,182 down-regulated genes were obtained (Figure 2A). Among these DEGs, there were 1,040 immune-related genes, of which 385 were up-regulated and 655 were down-regulated, respectively (Figures 2B,C).

### GO and KEGG Annotation of the DEGs

The results of GO functional annotation analysis of the immune-related DEGs showed that the most significantly enriched biological processes (BPs) included homophilic cell adhesion *via* plasma membrane adhesion molecules, extracellular structure organization, and extracellular matrix organization. The most significantly enriched cellular components (CCs) included collagen trimers, the endoplasmic reticulum lumen, and cell–cell junctions, and the molecular functions (MFs) included integrin binding, cytokine receptor binding, and cytokine activity (Figure 2D). KEGG pathway enrichment analysis for the immune-related DEGs showed that the significant pathways included the Rap1 signaling pathway, the JAK–STAT signaling pathway, transcriptional misregulation in cancer and proteoglycans in cancer (Figure 2E). Most of the above results were related to immunity.

### Construction of the Immune-Related Signature

**Figure 3:**
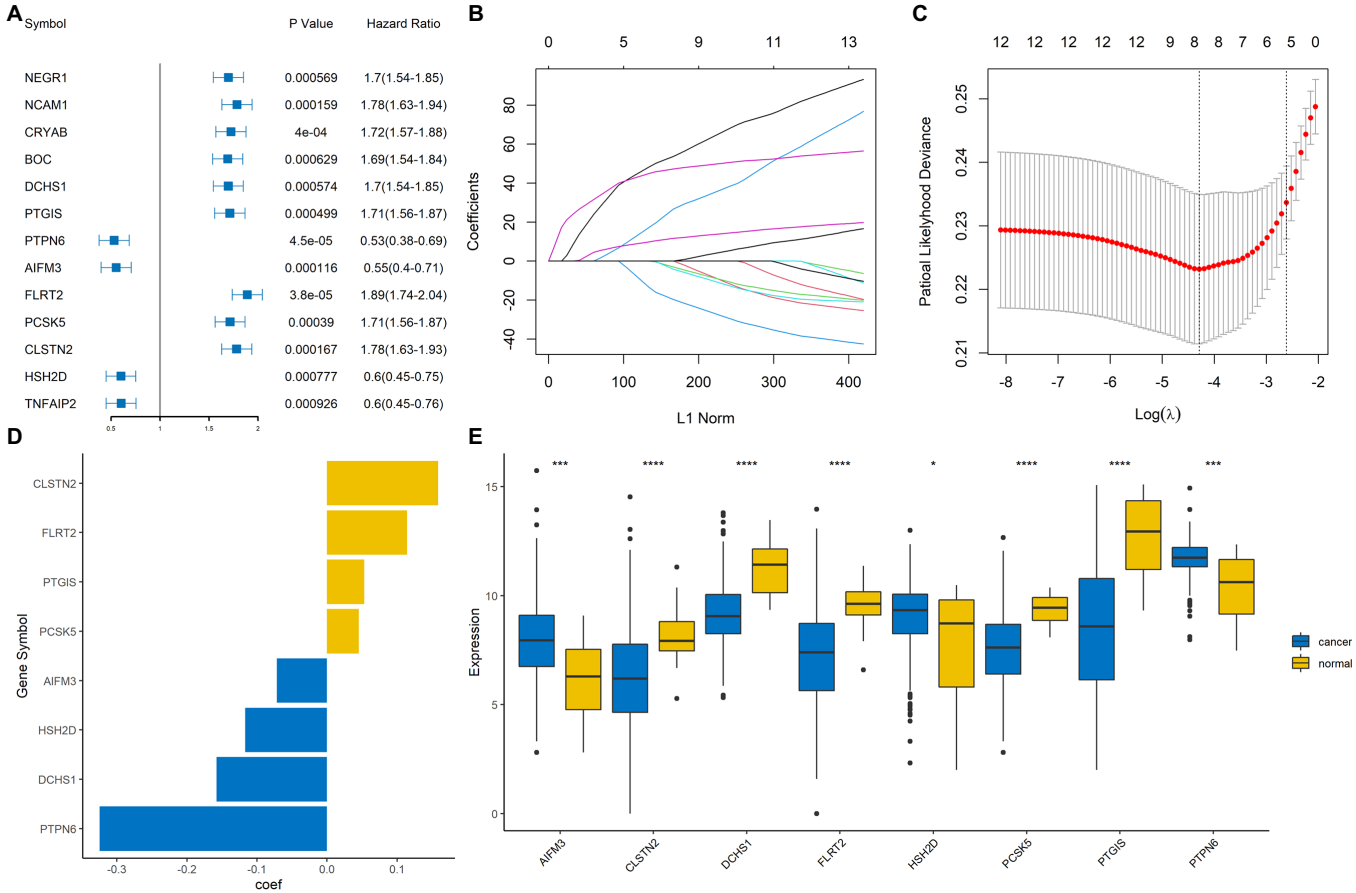
Construction of an immune-related signature model. **(A)** Forrest plot showing the hazard ratio and value of *p* for the candidate genes. **(B,C)** Least absolute shrinkage and selection operator (LASSO) Cox regression analysis identified eight immune-related signature genes that were most correlated with overall survival (OS). **(D)** Bar plot showing the coefficient value for the eight immune-related signature genes. **(E)** Boxplot showing the expression profile of the eight immune-related signature genes in tumor and normal tissues. ^****^, ^***^, and ^*^ represented *p* < 0.00001, *p* < 0.0001, and *p* < 0.05, respectively.

Univariate Cox regression based on the survival and gene expression data of BLCA patients was used to evaluate the prognostic value of the immune-related DEGs. In total, 13 immune-related DEGs were selected as candidate genes to construct the prognostic model with the criterion of value of *p*<0.001. A forest plot showing the value of *p* and hazard ratio of the candidate genes is shown in Figure 3A.

The BLCA cohort was randomly divided into training and testing sets at a ratio of 7:3. LASSO regression analysis was performed using the training data set to identify genes that were most significantly related to survival outcomes (Figures 3B,C). A total of eight signature genes were identified: DCHS1, PTGIS, PTPN6, AIFM3, FLRT2, PCSK5, CLSTN2, and HSH2D (Figure 3D); the coefficients of the signature genes are shown in Supplementary Table 1. Next, we investigated the expression profile of these eight genes in the BLCA cohort. The results indicated that AIFM3, HSH2D, and PTPN6 were significantly up-regulated, while DCHS1, PTGIS, FLRT2, PCSK5, and CLSTN2 were significantly down-regulated in tumor samples compared with normal samples (Figure 3E).

### A High-Risk Score Is Correlated With Poor Clinical Outcome in BLCA

**Figure 4:**
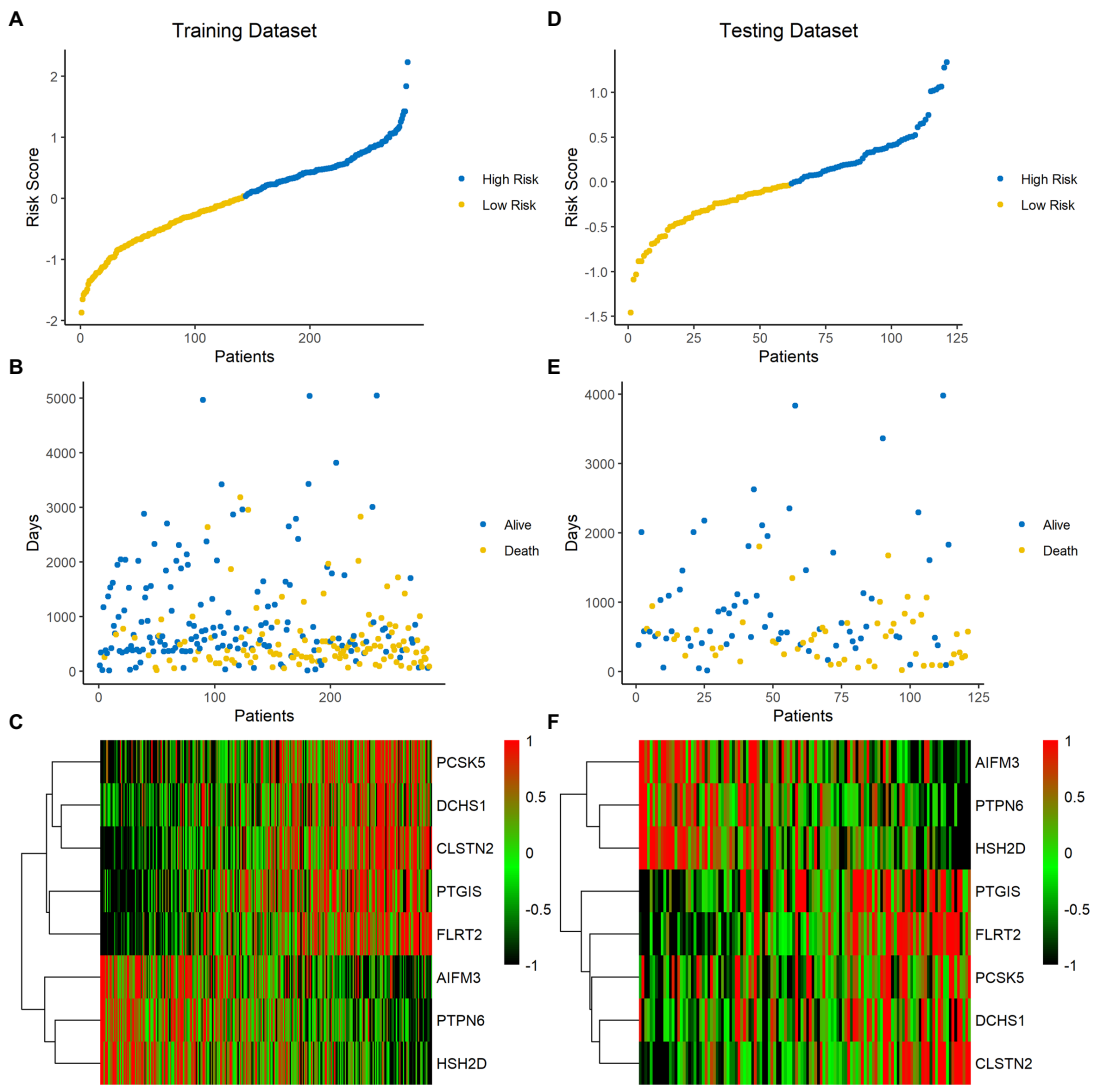
Analysis of the signature score in the training and testing sets. **(A–C)** Training set. **(D–F)** Testing set. The ranked dot plot indicates the risk score distribution in the training set **(A)** and testing set **(D)**. Scatter plot presenting the patients’ survival status in the training set **(B)** and testing set **(E)**. Heatmap showing the expression profile of the eight signature genes in BLCA patients from the training set **(C)** and testing set **(F)**.

**Figure 5:**
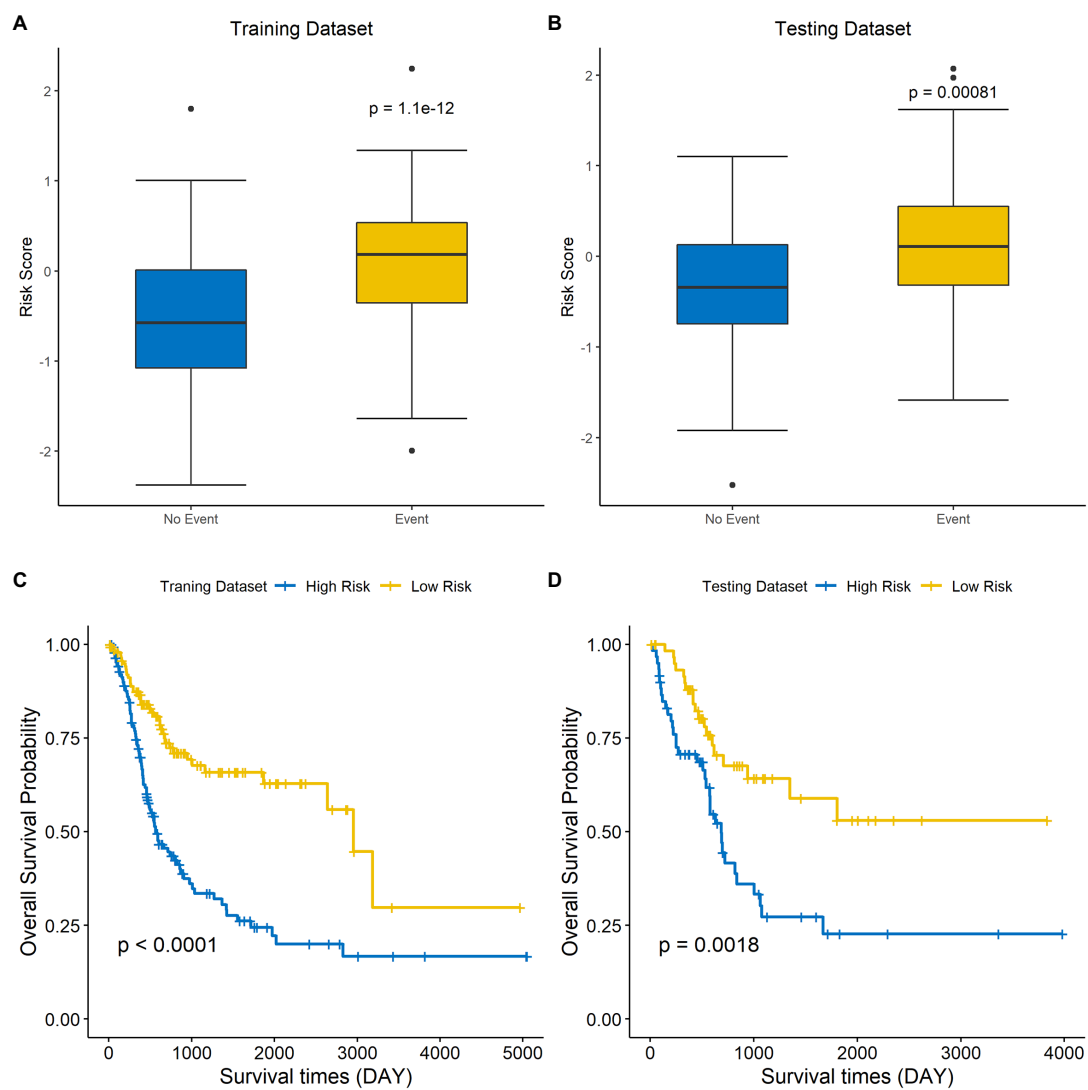
A low-risk score is correlated with better OS in BLCA. The differences in risk scores for patients with endpoint events *vs* nonendpoint events in the training set **(A)** and testing set **(B)**. Survival analysis of the high-risk and low-risk groups in the training **(C)** and testing sets **(D)**.

The risk score of each sample was calculated and ranked on the basis of the signature model in the training set (Figure 4A). The scatter plot represented the OS status of BLCA patients according to the risk score, and it suggested that the high-risk group had higher mortality than the low-risk group (Figure 4B). The expression profiles of the signature genes showed that tumors with higher risk scores tended to exhibit elevated PCSK5, DCHS1, CLSTN2, PTGIS, and FLRT2 levels, while those with lower risk scores tended to exhibit elevated AIFM3, PTPN6, and HSH2D levels (Figure 4C). The same analysis was performed using the testing set, and the results were consistent with those derived from the training data set (Figures 4D–F). Compared with the low-risk group, the high-risk group presented a significantly poorer clinical outcome in both the training (*p*<0.001, Figure 5A) and testing sets (*p*<0.001, Figure 5B). Then, the association between the risk score and OS was evaluated, and Kaplan–Meier analysis showed that the low-risk groups had a longer survival time than the high-risk group in both the training (Figure 5C, *p*<0.0001) and testing sets (Figure 5D, *p*=0.0018). These results demonstrated that the risk score was associated with OS and that a low-risk score predicts better survival outcomes for BLCA patients.

### Estimating the Degree of Each Immune Cell Infiltration and Predicting the Response to Immunotherapy

**Figure 6:**
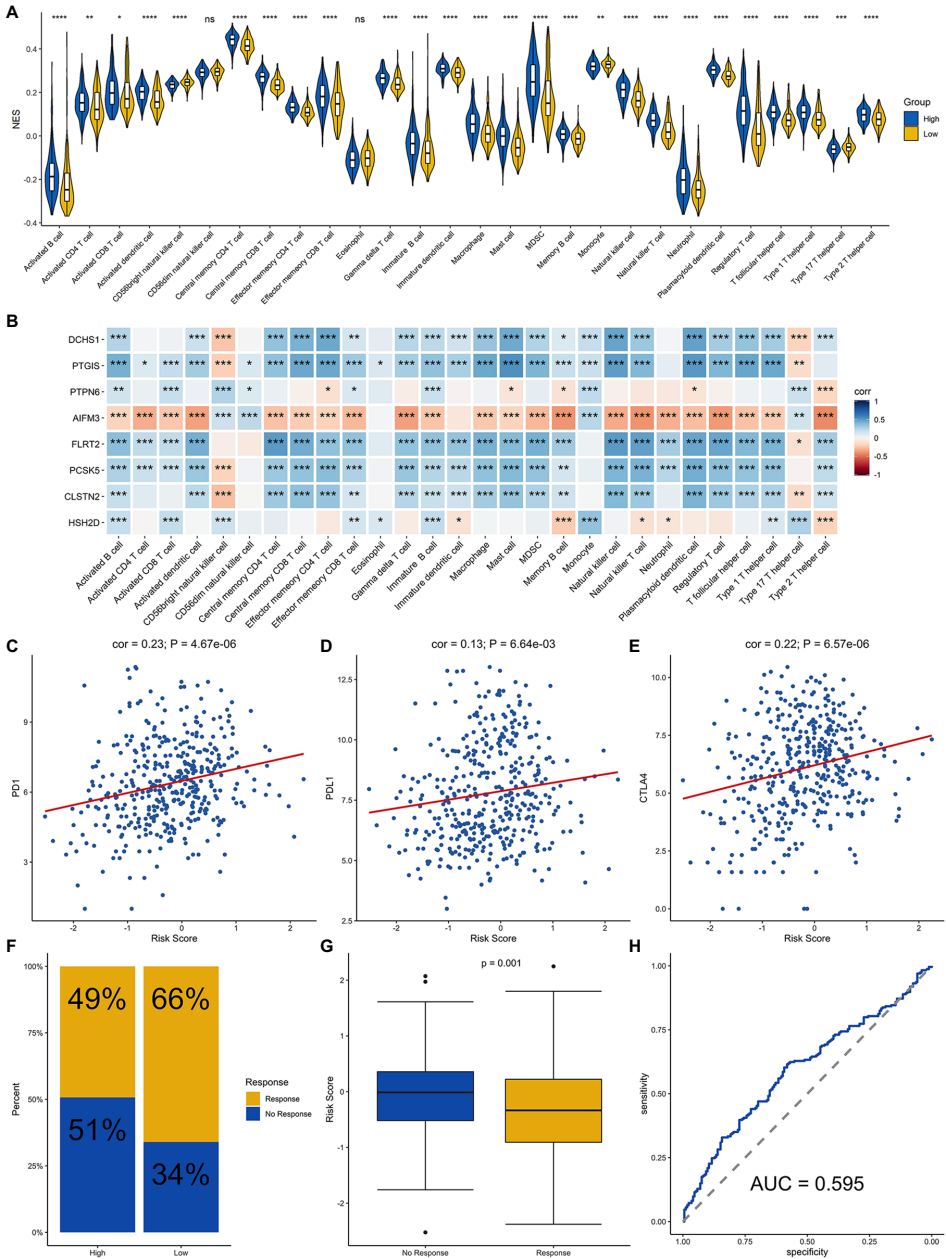
Analysis of immune cell infiltration and prediction of the response to immunotherapy in BLCA patients. **(A)** Infiltration level of immune cells in the high-risk and low-risk groups. **(B)** Correlation analysis for the eight signature genes and the infiltration level of the immune cells. Correlation analysis of the risk score and PD-1 **(C)**, PD-L1 **(D)**, and CTLA4 **(E)**. **(F)** Proportion of patients who responded to immunotherapy in the high-risk group and the low-risk group estimated by the TIDE algorithm. **(G)** Comparison of the risk score of the response group with that of the nonresponse group. **(H)** Receiver operating characteristic (ROC) curve for predicting the response to immunotherapy based on the risk score for BLCA. ^****^, ^***^, ^**^, ^*^ and ns represented *p* < 0.00001, *p* < 0.0001, *p* < 0.001, *p* < 0.05, and not significant, respectively.

Since the efficacy of immunotherapy depends on the level of immune cell infiltration, we analyzed the correlation between the level of immune cell infiltration and risk score. The results demonstrated that there were differences in the infiltration of most immune cells between the high-groups and low-risk groups, except for CD56dim NK cells and Eosinophil cells, which demonstrated that signature was significantly correlated with immune infiltration (Figure 6A). In addition, we explored the correlation between each of these eight genes and immune cell infiltration. DCHS1, PTGIS, FLRT2, PCSK5, and CLSTN2 were positively related to the infiltration almost all immune cells, and AIFM3 was negatively correlated with the infiltration of most immune cells (Figure 6B). Since the eight signature genes were significantly correlated with the level of immune cell infiltration, the correlation of the risk score and the immunotherapy response was explored. Firstly, the relationship between the risk score derived from the risk signature and the expression of immune checkpoint molecules was analyzed. The risk score was positively correlated with the expression of the checkpoint markers, PD-1 (Figure 6C), PD-L1 (Figure 6D) and CTLA-4 (Figure 6E), implicating the potential roles of the signature model in the response to immunotherapy in BLCA patients. The immunotherapy response of the BLCA cohort was estimated by the TIDE algorithm, and the ability of the model to predict the immunotherapy response was evaluated. Furthermore, we then calculated the immunotherapy response rate of samples in the high-risk and low-risk groups. Overall, 49 and 66% patients in the high-risk and low-risk groups, respectively, were predicted to respond to immunotherapy (Figure 6F). Patients were divided into responsive and nonresponsive groups, then their risk scores were calculated. The results indicated that the responsive group had significantly lower scores than the nonresponsive group (Figure 6G, *p*=0.001). An ROC curve was generated to determine the efficacy of the risk score in predicting the response to immunotherapy. The area under the ROC curve (AUC) was 0.595, suggesting that the immune-related signature model predicted the response to immunotherapy for BLCA with modest accuracy (Figure 6H). In summary, the eight-gene signature model was associated with immune cell infiltration and immunotherapy response in BLCA.

### Evaluation of the Model Performance

**Figure 7:**
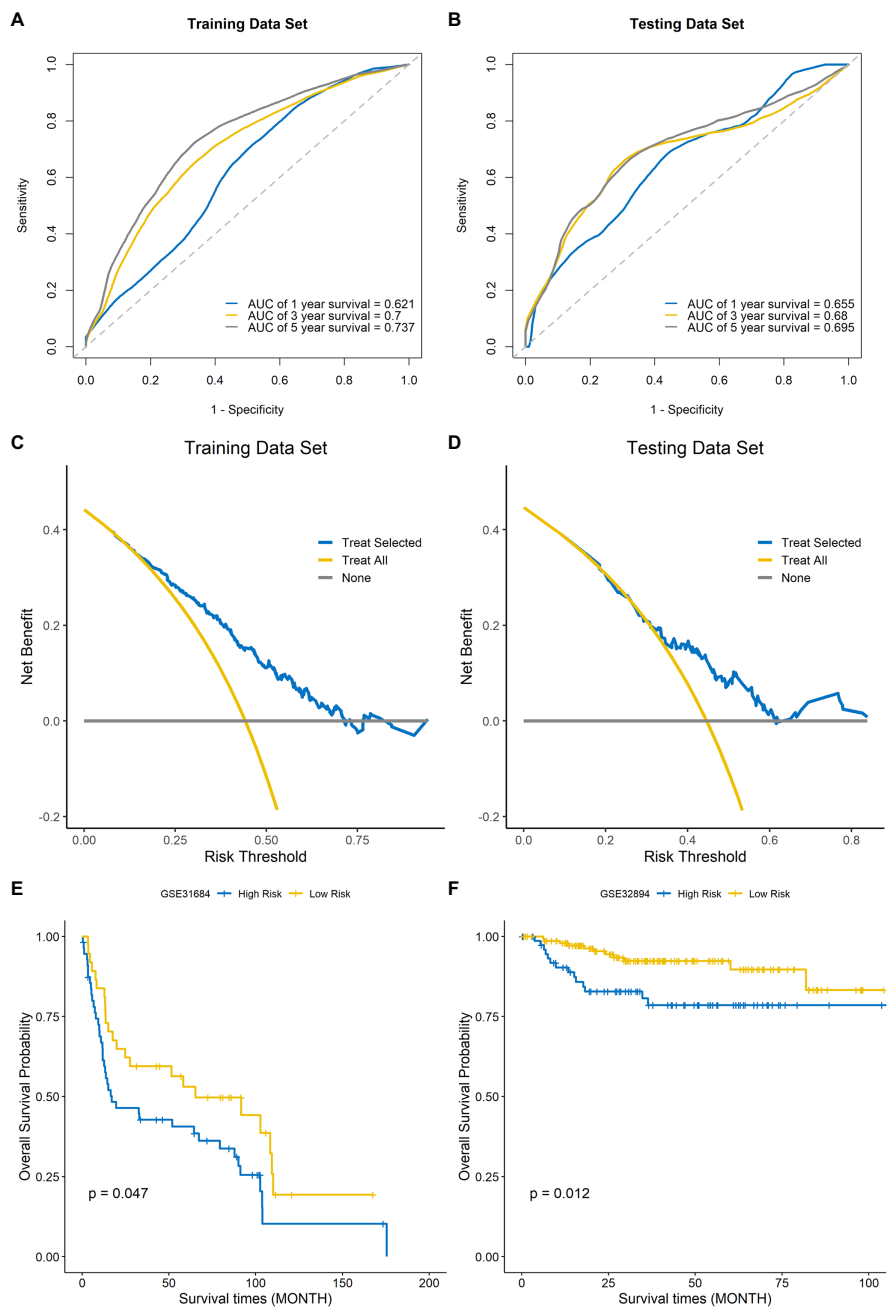
Evaluation of the performance of the signature model. ROC curves used for predicting the 1-, 3-, and 5-year ROC curves in the training set **(A)** and testing set **(B)**. Decision curve analysis of the training set **(C)** and testing set **(D)**. External validation of the signature model using the Gene Expression Omnibus (GEO) BLCA cohorts GSE31684 **(E)** and GSE32894 **(F)**.

Time-dependent ROC curves and AUCs were ploted to determine the prognostic values of the eight-gene risk score in the training and testing sets. The AUCs of the risk score for predicting 1-, 3-, and 5-year OS were 0.621, 0.7, and 0.737 in the training set (Figure 7A) and 0.655, 0.68, and 0.695 in testing set (Figure 7B). To compare the consistency of the model prediction with actual clinical outcomes, calibration curves for 1-, 3-, and 5-year OS in the training (Supplementary Figures 1A–C) and testing sets (Supplementary Figures 1D–F) were constructed. The results suggested that the calibration curves showed satisfactory agreement between the predicted and observed values for 1-, 3-, and 5-year OS. To determine the clinical usefulness of the risk signature, decision curve analysis in the training and testing sets was performed. Decision curve analysis showed that the risk scores offered a net benefit over the “treat-all” or “treat-none” strategy, which indicated that the model was clinically useful (Figures 7C,D). External validation was performed using the BLCA GEO database cohort (GSE31684 and GSE32894). All the samples were divided into high-risk and low-risk groups based on the optimal cutoff point of the risk score, then Kaplan–Meier analysis was performed. The results suggested that the prognosis of the low-risk group was better than that of the high-risk group in the BLCA cohorts GSE31684 (Figure 7E, *p*=0.047) and GSE32894 (Figure 7F, *p*=0.012). The results above indicate that this model had good predictive power in both the TCGA and other external cohorts.

## Discussion

Tumors arise as a result of the accumulation of genetic mutations (Williams and Stein, 2004), and a large number of point mutations and structural changes occur during the development of tumors (Stratton et al., 2009), which inevitably stimulates the production of corresponding tumor antigens and thus induces an immune response (Zhang and Zhang, 2020). In recent years, with in-depth research on tumor immunology and molecular biology, immunotherapy has provided a new direction for tumor treatment (Ma et al., 2021). A variety of immune checkpoint (PD-1, PD-L1, and CTLA-4) inhibitors (ICIs) are being used in the treatment of advanced BLCA, and clinical studies have shown that they are reliable in terms of safety and efficacy. However, not every BLCA patient benefits from immunotherapy (Fumet et al., 2020). Consequently, it is imperative to identify biomarkers that can predict patient response to immunotherapy. In the present study, we aimed to construct an immune-related DEGs model, to investigate the relationship between this model and patient prognosis as well as the immunotherapy response, and to assess the potential clinical applications of the model.

In the present study, we obtained 1,040 immune-related BLCA DEGs by analyzing the TCGA database; 385 were up-regulated and 655 were down-regulated. Thirteen immune-related DEGs with significant prognostic value were screened by a one-way Cox regression model (*p*<0.001). Subsequently, we used LASSO regression analysis to screen eight signature genes from the 13 candidate genes in the univariate Cox regression model. The eight genes were CLSTN2, FLRT2, PTGIS, PCSK5, AIFM3, HSH2D, DCHS1, and PTPN6. FLRT2 is involved in the development of ovarian and uterine carcinogenesis, and FLRT2 has been reported to be a tumor suppressor in breast and prostate cancer (Donninger et al., 2004; Santin et al., 2005; Wu et al., 2016; Bae et al., 2017). The MethHc database show that PTGIS has a high level of DNA methylation in BLCA. PTGIS is a HIF-1α target gene that plays a primary regulatory role in hypoxic tumor progression by activating the transcription of various oncogenes (Lu et al., 2018). PCSK5 is one member of the chymosin-like proprotein convertase family, which can regulate the cleavage and activation of the prestructural domain of TGFβ/bmp family members. PCSK5 plays a major role in the mouse skeleton and organogenesis (Szumska et al., 2017). AIFM3 express in a variety of tissues and aberrantly expressed in several cancers, widely. AIFM3 is a direct target of miR-210 and is associated with the proliferation of human liver cancer cells (Yang et al., 2012), and overexpression of AIFM3 predicts stronger proliferative and invasive behavior in breast cancer (Zheng et al., 2019). HSH2D is an important signaling molecule that can affect T cell activation (Oda et al., 2001; Lapinski et al., 2009). HSH2D inhibits the transcriptional activation of the IL-2 promoter, specifically at the RE/AP element of IL-2, which is regulated by CD28, and HSH2D expression contributes to methotrexate resistance in human T-cell acute lymphoblastic leukemia (Shapiro et al., 2004; Pegram et al., 2015). DCHS1 plays a significant role in innate immunity in the human kidney and bladder according to immunostaining studies, and DCHS1 also can participate in cell adhesion, growth, planar cell polarity and tissue pattern (Liang et al., 2019; Qureshi et al., 2020). PTPN6 is thought to be a signaling molecule that can regulate many of cellular processes, including cell growth, differentiation, oncogenic transformation and mitotic cycle, as well as can act as a tumor suppressor. PTPN6 may improve the chemotherapy efficacy and can be used in combination with blocking antibodies in immunotherapy; moreover, some studies suggest that PTPN6 may be an immune-related prognostic biomarker for BLCA (Shen et al., 2020).

There is growing evidence that the immune microenvironment, in which immune cells and molecules are important components, acts an important role in tumor development and the degree of immune cell infiltration is highly correlated with patient prognosis (Grivennikov et al., 2010; Seager et al., 2017). With the recent development of technologies such as RNA-seq, it is possible to systematically analyze the tumor microenvironment and the functional diversity of tumor-infiltrating immune cells, the sensitivity of patients to immunotherapy and the prognosis (Zhang and Zhang, 2019). In recent study, we constructed an eight-gene signature model. The risk scores were significantly associated with the infiltration level of immune cells. Furthermore, six of the eight signature genes showed a significant positive or negative correlation with the infiltration level of immune cells. We also assessed the correlation of the eight signature genes with the response to immunotherapy, and the results suggested that the risk score was significantly and positively correlated with the expression of the checkpoint markers, PD-1, PD-L1, and CTLA-4. The immune-related signature model predicted the response to immunotherapy for BLCA with good accuracy. We applied this signature model to evaluate the clinical data, and the results showed that patients in the low-risk group had better clinical outcomes than those in the high-risk group. We performed Kaplan–Meier analysis on the training set, and the results showed that the low-risk group had a longer survival time than the high-risk group (*p*<0.001). The above data suggest that this model has good clinical feasibility. We validated the accuracy and clinical usefulness of the signature model using several methods, including ROC curves for 1-, 3-, and 5-year OS and decision curve analysis. Finally, external validation of the model using the GEO BLCA cohorts further verified the prognostic ability of the model.

However, there were some limitations of this study. This study was retrospective, and further prospective studies are required to validate our findings, and in this study, only innate immunity genes were analyzed and adaptive immunity was not involved. In addition, some clinical characteristics, such as age and clinical stages, were not included in our model.

## Conclusion

In this study, we established an immune-related eight-gene signature model for BLCA, that could be used to predict the immune response and prognosis of BLCA patients.

## Data Availability Statement

The original contributions presented in the study are included in the article/Supplementary Material; further inquiries can be directed to the corresponding author.

## Author Contributions

TY made substantial contributions to the conception, design, interpretation, and preparation of the final manuscript. BD, JL, WS, SZ, YM, JS, and MZ participated in the coordination of data acquisition and data analysis and reviewed the manuscript. DL reviewed and revised the manuscript. All authors contributed to the article and approved the submitted version.

## Conflict of Interest

The authors declare that the research was conducted in the absence of any commercial or financial relationships that could be construed as a potential conflict of interest.

## Publisher’s Note

All claims expressed in this article are solely those of the authors and do not necessarily represent those of their affiliated organizations, or those of the publisher, the editors and the reviewers. Any product that may be evaluated in this article, or claim that may be made by its manufacturer, is not guaranteed or endorsed by the publisher.

## Footnotes

^1^ https://www.innatedb.ca/

^2^ http://tide.dfci.harvard.edu

## Supplementary Material

Supplementary Figure 1 Calibration curves for evaluating the accuracy of the model in predicting 1-, 3-, and 5-year OS in the training **(A–C)** and testing sets **(D–F)**.

Supplementary Figure 2 Analysis of immune cell infiltration in GEO BLCA cohorts GSE31684 **(A)** and GSE32894 **(B)**.

Supplementary Table 1 The coefficients of the signature genes.

Supplementary Table 2 The clinical information of TCGA BLCA data.

Supplementary Table 3 The clinical information of GSE32894.

Supplementary Table 4 The clinical information of GSE31684.
