# Supplementary figures and images for: Tumor Expression Profile Analysis Developed and Validated a Prognostic Model Based on Immune-Related Genes in Bladder Cancer
Source: Front Genet. 2021 Aug 27;12:696912. doi: 10.3389/fgene.2021.696912 (PMC8429908; doi:10.3389/fgene.2021.696912)

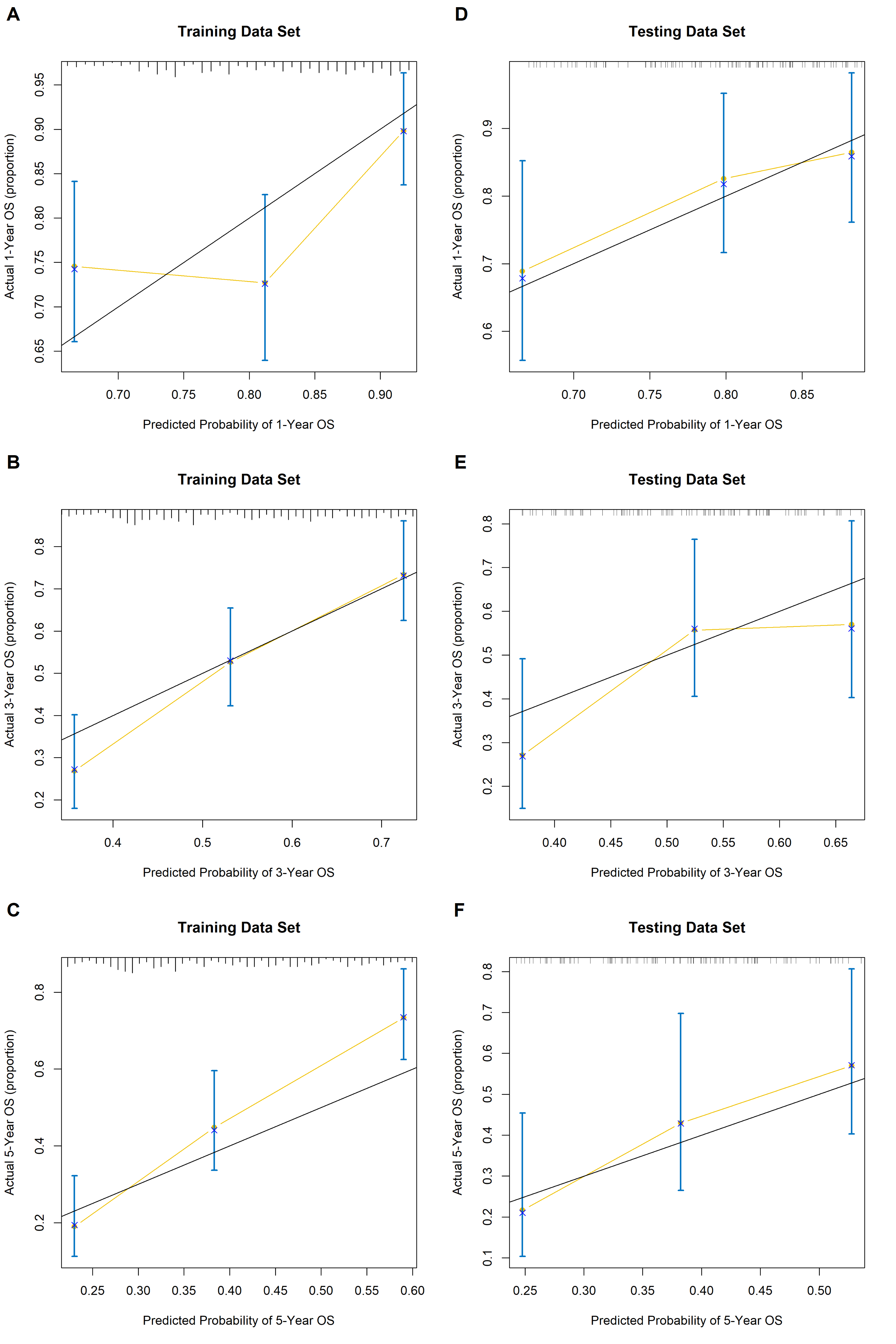

Supplement: Supplementary Figure 1 — Calibration curves for evaluating the accuracy of the model in predicting 1-, 3-, and 5-year OS in the training (A–C) and testing sets (D–F). [file Image_1.TIFF]

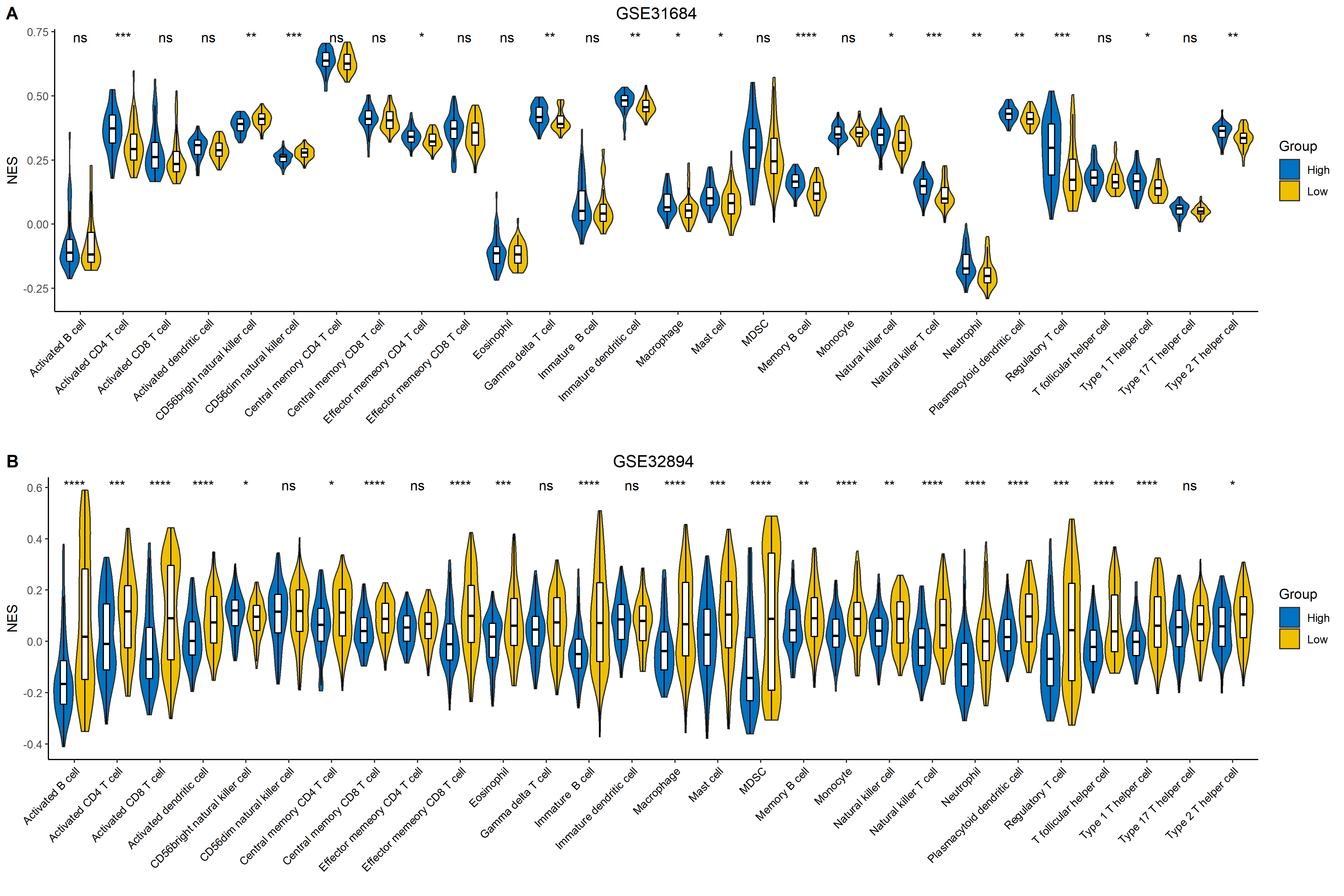

Supplement: Supplementary Figure 2 — Analysis of immune cell infiltration in GEO BLCA cohorts GSE31684 (A) and GSE32894 (B). [file Image_2.TIF]
