# Supplementary material for: Tumor Expression Profile Analysis Developed and Validated a Prognostic Model Based on Immune-Related Genes in Bladder Cancer
Source: Front Genet. 2021 Aug 27;12:696912. doi: 10.3389/fgene.2021.696912 (PMC8429908; doi:10.3389/fgene.2021.696912)
Supplement: Supplementary Table 1 — The coefficients of the signature genes. [file Table_1.DOCX]

| symbol | Coefficient |
| --- | --- |
| DCHS1 | -0.15744481 |
| PTGIS | 0.05310281 |
| PTPN6 | -0.32469170 |
| AIFM3 | -0.07170157 |
| FLRT2 | 0.11454092 |
| PCSK5 | 0.04565584 |
| CLSTN2 | 0.15854682 |
| HSH2D | -0.11663360 |
