# Supplementary material for: Tumor Expression Profile Analysis Developed and Validated a Prognostic Model Based on Immune-Related Genes in Bladder Cancer
Source: Front Genet. 2021 Aug 27;12:696912. doi: 10.3389/fgene.2021.696912 (PMC8429908; doi:10.3389/fgene.2021.696912)
Supplement: Supplementary Table 2 — The clinical information of TCGA BLCA data. [file Table_2.DOCX]

| Variable | N | TCGA, N = 448^1^ |
| --- | --- | --- |
| **sex** | 448 |  |
| female |  | 121 (27%) |
| male |  | 327 (73%) |
| **age** | 448 |  |
| <60 |  | 98 (22%) |
| >=60 |  | 350 (78%) |
| **stage** | 448 |  |
| I |  | 3 (0.7%) |
| II |  | 136 (30%) |
| III |  | 159 (35%) |
| IV |  | 148 (33%) |
| not reported |  | 2 (0.4%) |
| **stage_T** | 416 |  |
| T0 |  | 1 (0.2%) |
| T1 |  | 4 (1.0%) |
| T2 |  | 126 (30%) |
| T3 |  | 217 (52%) |
| T4 |  | 67 (16%) |
| TX |  | 1 (0.2%) |
| **stage_M** | 445 |  |
| M0 |  | 219 (49%) |
| M1 |  | 11 (2.5%) |
| MX |  | 215 (48%) |
| **stage_N** | 442 |  |
| N0 |  | 262 (59%) |
| N1 |  | 52 (12%) |
| N2 |  | 84 (19%) |
| N3 |  | 7 (1.6%) |
| NX |  | 37 (8.4%) |
| ^1^n (%) | | |
