# Supplementary material for: Tumor Expression Profile Analysis Developed and Validated a Prognostic Model Based on Immune-Related Genes in Bladder Cancer
Source: Front Genet. 2021 Aug 27;12:696912. doi: 10.3389/fgene.2021.696912 (PMC8429908; doi:10.3389/fgene.2021.696912)
Supplement: Supplementary Table 3 — The clinical information of GSE32894. [file Table_3.DOCX]

| Variable | N | GSE32894, N = 308^1^ |
| --- | --- | --- |
| **sex** | 308 |  |
| F |  | 80 (26%) |
| M |  | 228 (74%) |
| **age** | 308 | 71 (63, 79) |
| **stage** | 308 |  |
| T1 |  | 97 (31%) |
| T2 |  | 82 (27%) |
| T2a |  | 1 (0.3%) |
| T2b |  | 2 (0.6%) |
| T3 |  | 1 (0.3%) |
| T3b |  | 6 (1.9%) |
| T4a |  | 1 (0.3%) |
| Ta |  | 116 (38%) |
| Tx |  | 2 (0.6%) |
| ^1^n (%); Median (IQR) | | |
