# Supplementary material for: Tumor Expression Profile Analysis Developed and Validated a Prognostic Model Based on Immune-Related Genes in Bladder Cancer
Source: Front Genet. 2021 Aug 27;12:696912. doi: 10.3389/fgene.2021.696912 (PMC8429908; doi:10.3389/fgene.2021.696912)
Supplement: Supplementary Table 4 — The clinical information of GSE31684. [file Table_4.DOCX]

| Variable | N | GSE31684, N = 93^1^ |
| --- | --- | --- |
| **sex** | 93 |  |
| female |  | 25 (27%) |
| male |  | 68 (73%) |
| **stage** | 93 |  |
| T1 |  | 19 (20%) |
| T2 |  | 55 (59%) |
| T3 |  | 10 (11%) |
| T4 |  | 1 (1.1%) |
| Ta |  | 8 (8.6%) |
| **age** | 93 | 69 (62, 75) |
| ^1^n (%); Median (IQR) | | |
